# The Institutional Worker

**Published:** 1913-05-03

**Authors:** 


### INSTITUTIONAL WORKER

**Being a Special Supplement to "The Hospital"**

### OUR BUREAU OF INFORMATION.

### Rules for Correspondents.

1. Every letter, on whatever subject, must be accompanied by the coupon to be cut from the back cover (inside page) of The Hospital, current issue, and must contain the name and address of the correspondent with pseudonym for publication if desired.
2. Replies by post cannot be given, save under exceptional circumstances at the Editor's discretion.
3. Letters from Approved Homes in reply to special needs published in the Bureau should state terms and full particulars, and be sent prepaid under cover to the Editor of the Bureau with name written across coupon for identification.
4. Proprietors of Homes which have not yet been entered on the List of Approved Homes, but have spare accommodation likely to suit special needs, are invited to write for an application form for registration. The fee for registration, which includes two announcements of the Home in the Bureau and other privileges, is 10s.
5. All communications to be addressed to the Editor of The Hospital, 28 Southampton Street, Strand, London, W.C., and marked "Bureau of Information."

### Invitation to Readers.

**The Editor is prepared to make known without charge the needs of any who may be sick or in difficulties, and to guide them in making choice of Homes for treatment or convalescence. Reduced terms can often be arranged with Homes on the Approved List for those unable to pay full fees. Queries are specially invited from those who are engaged in any kind of philanthropical work. A new edition of the leaflet describing the purposes of the Bureau of Information is now ready and will be sent free of charge on application.**

### Approved Homes.

**London** —136 Thornbury Road, Osterley Park, W. Principal: Miss R. G. Campbell, certificated nurse and masseuse. A new private Home for medical, surgical, and maternity cases, and for cases requiring massage and rest treatment. Accommodation also for delicate children, and for one case requiring open-air treatment. Highly recommended. Terms from £2 2s. to £6 6s.

**St. Neots.**—Berkley Villas, Eynesbury. Principal: Miss Rose E. Pearmain. A comfortable home for invalids or convalescents. Terms from 25s. weekly.

**Provincial.** — Private Home for neurasthenic, slight mental, and chronic cases. Weir-Mitchell, massage, and electrical treatment. Under constant medical supervision by specialist. Terms from six guineas a week. Applications should be made to "X. Y.," The Hospital Bureau of Information.

### Nursing Home, San Remo.

Doubtless you refer to the Maternity Charity and District Nurses' Home, Plaistow, London, E. The maternity training here is excellent, though, of course, there are many other good schools. For trained nurses the fees for courses of four, six, nine, and twelve months are £15, £25, £30, and £36 respectively. A twelve-months' training in midwifery and general district nursing is strongly recommended; or, where this is impossible, nine months. The fee for the C.M.B. Examination is £1 1s. The usual period for fever training is two years, though it is possible to take a shorter course. You might apply to the London Fever Hospital, Islington, N., or to the Clerk of the Metropolitan Asylums Board, Victoria Embankment, London. There are, of course, many provincial institutions. As you propose to come to London, it would possibly be best for you to obtain your training in massage at the National Hospital for the Paralysed and Epileptic, Queen Square, Bloomsbury; you should apply to the Matron. The fee for massage only is £10 10s., and £3 3s. extra if electricity be taken as well. You might also apply to the Incorporated Society of Trained Masseuses, 12 Buckingham Street, Strand, London, W.C.; they will give you valuable advice upon this matter.—"E. C. G. W."

### Massage Training.

Kindly read the rules and observe them; we cannot give postal replies. The examination conducted twice yearly by the Incorporated Society of Trained Masseuses, 12 Buckingham Street, Strand, London, W.C., is the recognised one. This institution has a registry for members, and advises them upon professional difficulties.

### The Mentally Defective.

The National Institutions for Persons Requiring Care and Control undertake the permanent care of those mentally-defective children who are unable to benefit by the ordinary means of education and yet are not of so low a mental grade as to be properly certifiable under the Idiots Act. Cases are not received at these institutions for temporary care, as the authorities consider that the mentally defective of both sexes, whilst being capable of improvement, never become fit

There is an increasing number of hospitals, both in London and the provinces, which train nurses in this work, and full particulars of these may be obtained of the institution referred to. If you decide to come to London, we would advise you to apply to the Matron, National Hospital for Paralysed and Epileptic, Queen Square, Bloomsbury. The fee for a course of training in massage only is £10 10s., and £3 3s. extra for electricity.—"L. S."

### Help for Domestic Servant.

It is possible that one of the following societies may be able to help "A. W.," whose condition of health renders her unfit for domestic service of an arduous character, (*a*) The Domestic Servants' Benevolent Institution, 32 Sackville Street, Piccadilly, London, W., or (*b*) the Metropolitan Association for Befriending Young Servants, 66 Denison House, Vauxhall Bridge Road, London, S.W.—"A. E. B."

### Distressed Family of Medical Practitioner.

The case you mention is one where application for help should be made to the Royal Medical Benevolent Fund. This institution makes grants of money to the widows and orphans of medical practitioners, as well as to distressed practitioners themselves. It also provides annuities (generally of the value of £20) to such cases after they have reached the age of sixty years. The benefits of the Fund are conferred upon all deserving cases, without restriction. Full inquiries are made into the circumstances of each case before relief is given, and no canvassing is required. Application should be made to the Hon. Secretary, at 11 Chandos Street, Cavendish Square, W.—"S. P."

### Smoke Nuisance.

The complaint is by no means unreasonable, and most local authorities reserve power under their bye-laws to prosecute those who are responsible for a nuisance of this character. You can quite easily overcome the trouble by paying a little attention to the stoking. For the furnace of a vertical boiler such as you describe you will find it best to keep the surface of the burning fuel concave (or saucer shaped); this can easily be done by placing the fuel at the circumference instead of in the centre of the furnace. If this plan be followed out, with the exception during a period of a minute or so after stoking, scarcely any smoke will be visible at the top of the stack, for most of it is through this arrangement drawn toward the centre of the furnace and consumed. No doubt there are many capable engineers and stokers who can give practical hints upon this difficulty, and the columns of the Bureau are open for an expression of their opinions.—"Stoker."

### Prevention of Damage to Ward Walls.

The best way to overcome the difficulty you complain of is to attach a buffer with a rubber head to each side of the head of the bedstead, on a level with the spring mattress. Most modern bedsteads have these attachments; any able mechanic would, however, devise something suitable for yours. The only alternative to this is to fix a strip of beading on the floor, at a sufficient distance from the wall; but the objection to this is that it offers lodgment for dirt and dust, and interferes with the process of polishing.—"Cottage Hospital."

### Reformation of Young Women.

The Royal Female Philanthropic Society may be able to assist you. One of the objects of this Society is to assist young women who are convicted and imprisoned for a first offence, or who are discharged from service for dishonesty. Women are received into the Society's Home for a period of two years, where they are instructed in every branch of household work. Admission is by subscriber's recommendation, although a non-subscriber is permitted to recommend upon contributing one guinea toward the funds of the Society; a payment of 5s. for maintenance is required in addition, but in very special circumstances a smaller sum is accepted. Full particulars may be obtained upon application to the Secretary, 53 Great Church Lane, Hammersmith.—"P. S."

### Egg Flip.

Beat half an ounce of powdered sugar and the yolks of two eggs together, adding eight tablespoonfuls of brandy and eight tablespoonfuls of cinnamon water previously mixed together.—"Invalid."

### How to Become a Hospital Nurse.

Please read the rules and observe them. You are somewhat too young, at the age of twenty-one years, to enter for training at a general hospital. We would advise you to obtain and read the book, "How to Become a Nurse," by Sir Henry Burdett, price 2s. 3d. post free, published by The Scientific Press, Ltd., 28 and 29 Southampton Street, Strand, London, W.C. This will give you the fullest information upon the subject.—"M. H."

to compete on equal terms with normal people. One of these institutions, the Stoke Park Colony, Stapleton, Bristol, affords accommodation for 428 persons; here are admitted males between the ages of two and ten years, and females between the ages of two and twenty-five years. The charge for the maintenance of each case is 10s. 6d. per week. The children are trained in domestic and industrial occupations, which enable them ultimately to take some small place in the Colony. Full particulars may be had on application to the Warden, 14 Howick Place, Victoria Street, Westminster, S.W.—"Guardian."

### Admissions Department of a Hospital.

The question of the admission of patients gives rise to some difficulty at many of the smaller hospitals, and although a great deal depends upon the design of the admission department, as well as the conditions of service, much can be done to improve the state of things you complain of by the tactful exercise of personal supervision. In no circumstance is there any excuse for delay in the admission of cases, and the absence of an official can only be attributed to faulty management. In the large hospitals the superintendent is primarily responsible for the admission or refusal of cases, but in the smaller institutions this duty is largely left to the house surgeon, who as a rule is not a permanent official, and cannot therefore be expected to have a knowledge of the locality in which the hospital he serves is situated, or of its needs. These features are often the cause of serious trouble, and are indications that for the efficient management of this department, co-operation between the house surgeon and a permanent administrative official is essential.—"Worried."

### Institution Facts and Figures.

### Question for May.

During a period of four weeks in the winter months state what was the quantity of gas consumed in your institution for purposes other than illuminating, its approximate cost, the quantity of each kind of fuel used and its cost, deducting the cost of fuel for laundry purposes (if any).

In answering this question the following particulars should be given: (1) Number of wards and size of each (in beds); (2) total number of coal fires (*a*) in the wards, (*b*) in the rest of the building; (3) the number of gas-cooking and other stoves and sterilisers; (4) the name of the heating and hot-water supply service.

In the following month contributors will be asked to give fuller particulars upon this subject by describing the character of the buildings, apparatus, etc.

---

The figures must be the result of actual observation and computation, not estimates. The best answers will be published, and for each contribution considered by the Editor to be of sufficient interest for publication payment of Five Shillings will be made.

Rules.

The following rules must be observed by all those answering the Facts and Figures Questions:—

1. Answers must be written on one side of the paper. They must bear the name and address of the sender and be accompanied by coupon to be cut from the back cover (inside page) of the current issue of The Hospital. A pseudonym must be chosen if the name is not to be published.
2. Answers must be addressed to the Editor of The Hospital, 28-29 Southampton Street, Strand, London, W.C.; they must be marked in the left-hand corner "Facts and Figures," and must be received before the end of the month.

### Hospital for Spinal Disease.

Please read the rules, and comply with them. We cannot give postal replies. The Royal National Orthopædic Hospital, 234 Great Portland Street, W., undertakes, among others, the treatment of spinal cases. The out-patient department is open at 1.30 p.m. each day except Saturday.—"D. J. S."

### Surgeons' Rubber Gloves.

There is now a large number of different makes of rubber gloves in the market, some of which are of an inferior quality. There is no general rule that we can suggest that will assist you in the matter of selection. If you have had no experience of the particular kind in question, it is desirable that you should examine them to see that the shape is satisfactory, as in some gloves the fingers are too short, and the thumb is badly placed. You should also insist on boiling a sample of the gloves; you will be able to judge by the result whether the quality be good or otherwise.—"Dispenser."

### Out-Patient (Electrical) Departments.

Many London hospitals have electrical departments for the treatment of out-patients; St. Bartholomew's, West Smithfield, would probably be the most convenient for those coming from Homerton (to Broad Street Station). Treatment is given to out-patients in the electrical department of this institution on Mondays, Tuesdays, Thursdays, and Fridays, at 1.30 p.m.—"L. S."

### The Editor's Letter-Box.

### Communications have been received and attended to from:—

E. Newmarch, E. Cole, Miss Green, Miss E. Purton, Miss Hilda Wood, Mrs. Hewitt, "X. Y.," Miss R. G. Campbell, and Miss Owen. Communications received from M. Day, Nurse Knight, and Mrs. E. J. LeMar are being attended to.

### Editor's Notices.

**Contributions:**

Contributions should be written, or preferably typed, on one side of the paper only, and all articles sent in are accepted upon the distinct understanding that they are forwarded to THE HOSPITAL only.

The Editor cannot undertake to return MSS. not used, but when a stamped directed envelope is enclosed the MSS. may be returned if a special request is made.

Accepted articles and paragraphs of news will be paid for after publication at the scale rate.

**Address:**

To prevent delay all contributions and letters on editorial business must be addressed exclusively to the Editor, "The Hospital," 29 Southampton Street, Strand, London, W.C. It is important that this regulation shall be strictly observed.

**Correspondence:**

Correspondence on all subjects is invited. The name and address of correspondents must be given as a guarantee of good faith, but not necessarily for publication.

**Special Articles:**

Special articles are invited, and questions, inquiries, and paragraphs upon all matters relating to the work, administration, and management of general, special, mental, fever, cottage and convalescent hospitals, sanatoria, homes, institutions, societies and organisations for the treatment or care of the sick, injured and dependants of all classes. Every matter affecting the interests and welfare of the staff of all grades working in these institutions will receive special consideration.

**Administrative Medicine, Research and Items of News:**

Special terms will be paid for approved articles on subjects in this wide field by experts who have made some section of it their special study and interest. We wish to give prominence to every movement tending to advance accuracy of diagnosis, efficiency in treatment, and the development of modern methods to eradicate disease and promote the welfare of the suffering.

**A Bureau of Information:**

We invite inquiries and applications for information and help in providing for that numerous class of sufferers who require special care or house-room which they cannot obtain in their own homes or secure for themselves. We cannot, however, prescribe, or recommend practitioners.

**Books for Review:**

Publishers are particularly requested to send advance proofs of any new books of importance whenever possible, as the Editor has made arrangements to publish immediate reviews on a new plan.

**Photographs, Plans, Blocks, and Illustrations:**

It is requested that wherever possible MSS. may be accompanied by illustrations in any of the above forms. The name of the sender and of the article to which it belongs should be written on the back of each photograph, drawing, or block for purposes of identification.

**Local Papers:**

Newspapers containing reports or news paragraphs should be marked and addressed to the Sub-Editor.

**The Coupon System:**

A coupon will be found at the bottom of the third inside page of the cover each week. This coupon must be attached to every question or inquiry to which an answer is desired in THE HOSPITAL.

### Are You a Subscriber?

THE ever-increasing circulation of THE HOSPITAL often renders it a matter of some difficulty to procure a copy of the current issue at the local newsagents. By sending a subscription to the Journal either through a bookseller or direct to the Publishing Offices you guard against the contingency of THE HOSPITAL being sold, and ensure a copy reaching you almost immediately upon publication.

When seeking a post this is a great help, since it enables you to send in your application without delay, an advantage which may secure you the appointment. In addition to this, as a subscriber, no matter how many times you may change your address, or in what part of the country you may be at the moment, a postcard will ensure that you receive THE HOSPITAL on the same day and at the same hour as when at home.

**Subscriptions** may begin at any time, and are payable in advance. Cheques and Post-Office Orders should be crossed London County and Westminster Bank, Covent Garden Branch, and made payable to the Manager of THE HOSPITAL.

RATES OF SUBSCRIPTION (payable in advance).

| | | |
|---|---|---|
| **UNITED KINGDOM.** | | |
| Three Months (including Postage) | | 2s. 0d. |
| Six Months | do. | 4s. 0d. |
| Twelve Months | do. | 6s. 6d. |
| **FOREIGN AND COLONIAL.** | | |
| Three Months (including Postage) | | 2s. 6d. |
| Six Months | do. | 5s. 0d. |
| Twelve Months | do. | 8s. 8d. |

*SUBSCRIPTION FORM.*

*Please send me* THE HOSPITAL *for..............months, commencing with your issue of.............................., for which please find enclosed............................*

*Name*..................................................

*Address* ..................................................
..................................................

*Date*....................................

*To* .................................... *Newsagent.*

### Manager's Notices.

**Letters relating to the publication, sale, and advertising departments of "The Hospital" must be addressed to The Manager, "The Hospital," The Hospital Building, 28 and 29 Southampton Street, London, W.C.**

**Advertisements:**

To ensure insertion, all advertisements for THE HOSPITAL must reach the Manager not later than Wednesday morning in each week. For scale of charges see pages v and 4.

**Remittances:**

**It is especially requested that remittances be made by Cheque or Postal Order, and in halfpenny stamps only when the amount is under sixpence.**

